# The impact of COVID-19 pandemic in tuberculosis diagnosis in sub-Saharan Africa: data from DREAM program in Mozambique

**DOI:** 10.4314/ahs.v24i2.11

**Published:** 2024-06

**Authors:** Fausto Ciccacci, Kanyza Ibraimo, Alberto Sineque, Susanna Ceffa, Zita Sidumo, Stefano Orlando, Cristina Marazzi

**Affiliations:** 1 Unicamillus, Saint Camillus International, University of Health Sciences, Rome, Italy; 2 DREAM program, Community of Sant'Egidio, Beira, Mozambique; 3 DREAM program, Community of Sant'Egidio, Maputo, Mozambique; 4 DREAM program, Community of Sant'Egidio, Rome, Italy; 5 Department of Biomedicine and Prevention, University of Rome Torvergata, Rome, Italy; 6 LUMSA University, Rome, Italy

**Keywords:** COVID-19 pandemic, tuberculosis diagnosis, sub-Saharan Africa, Mozambique

## Abstract

**Background:**

TB is a global emergency, COVID-19 reversed the trend in TB mortality reduction to 2017 levels. Mozambique is one of the highest-burden countries with 368 new cases per 100.000 population in 2020.

**Objectives:**

This analysis aims to evaluate a TB diagnostic service in two Mozambican cities before and during the COVID-19 pandemic.

**Methods:**

We reviewed routine activity data from two laboratories in Mozambique (Maputo and Beira) in the period 01/2018–08/2022. GeneXpert test was prescribed based on clinical suspicion. Data about the number of tests, results, and rifampicin resistance were collected.

**Results:**

In the period 3,071 tests were conducted: 391 positive, and 32 rifampicin resistant. The number of positive samples was higher in Beira (20.2% vs 5%, OR 4[3.1-5.2]).

In Maputo, we observed a higher percentage of rifampicin-resistant samples (13.2%vs7%, OR 0.5[0.2-1.1]), but the overall prevalence of rifampicin resistance was higher in Beira (14.1‰vs6.6‰, OR 2.1[1.0-4.5]).

In 2020 and the first semester of 2021 a reduction in activity was observed, but positivity rates remained stable, with a slight increment starting in 2020.

**Conclusions:**

Our data confirm the impact of the COVID-19 pandemic on TB diagnostic services but also highlight possible benefits in terms of diagnostic appropriateness in clinical centers.

## Introduction

Tuberculosis (TB) was declared a global emergency by World Health Organization (WHO) in 1993 1. Since then, many efforts have been made to reduce its impact. TB mortality has been constantly reducing since 2005; in 2020 a 7% increment in TB deaths has been registered, for the first time in 15 years^2^. According to the WHO Global Tuberculosis Report 2021, such an increment is imputable to disruption in TB diagnostics and therapies due to the SARS-CoV-2 pandemic. COVID-19 reversed the positive trend in TB mortality reduction back to 2017 levels. African countries are historically burdened by TB, with the highest incidence rates in the world; 16 out of 30 high-burden countries for TB are in Sub-Saharan Africa, and 23/30 if we consider HIV-asociated TB. Despite the increment in mortality, TB notifications were reduced in most of the high-burden countries in 2020 and 2021, due to reduced diagnostic activity in the COVID-19 era^2^. The decline in TB diagnosis was much more modest in African countries.

Mozambique is one of the highest-burden countries, with 368 new TB cases per 100.000 population in 2020, around one out of three of them being HIV positive^2^. Despite high prevalence rates, Mozambique is one of the 6 high burden countries that achieved the milestone of 35% mortality reduction between 2015 and 2020, as expected by the End TB Strategy^3^.

This analysis aims to evaluate a TB diagnostic service in two Mozambican cities before and during the COVID-19 pandemic.

## Methods

We reviewed routine activity data from two laboratories in Mozambique: Maputo and Beira. The two laboratories are managed by the DREAM program, run by the Community of Sant'Egidio. DREAM program is a health program working in collaboration with the Ministry of Health, implementing a wide range of health services free of charge for the population, including TB diagnosis and treatment^4^-^7^. The two laboratories provide a large number of tests, including TB diagnosis. Tb diagnostic services in the two laboratories are carried out through the GeneX-pert® IV (Cepheid, Sunnyvale, US), which is a four-module PCR instrument, using either Xpert MTB/RIF and Xpert MTB/RIF Ultra assays according to availability. In particular, in Beira, the Idai cyclone destroyed part of the laboratory in March 2019, hence since 2020 Xpert MTB/RIF Ultra was used. Tb testing is performed using sputum samples, as per the manufacturer's instructions. The sputum samples are collected through the self-collection approach, by patients, following national recommendations and guidelines.

GeneXpert test was prescribed by physicians in health centers referring to the two laboratories, located in the same cities. Patients suspected of having TB, either due to positivity to the WHO clinical symptom screening or clinical suspicion, underwent GeneXpert testing^8^,^9^.

We reviewed the TB diagnostic activity of the two laboratories in the period January 2018 – August 2022. Data about the number of tests executed with positive and negative results and possible rifampicin resistance detected were collected. The positivity rate was calculated as the percentage of positive tests among the whole number of tests executed; the rifampicin resistance rate was calculated as the percentage of positive tests that tested rifampicin-resistant, while rifampicin resistance prevalence rates were calculated as the number of rifampicin resistance test per 1,000 tested executed.

## Results

In the period, the two laboratories conducted 3,071 GeneXpert tests; 391 samples resulted positive, and 32 were rifampicin resistant. Table 1 resumes the main results. The number of tests executed in the two laboratories in the period was similar, however, the number of positive samples was higher in Beira, and the difference was statistically significative (20.2% vs 5%, OR 4 [3.1-5.2]). In Maputo laboratory, we had a higher percentage of rifampicin-resistant samples, but the difference was not significative (13.2% vs 7%, OR 0.5[0.2-1.1]), however when considering the overall prevalence of rifampicin resistance among the tested samples, we observed a higher prevalence in Beira (14.1 ‰ vs 6.6 ‰, OR 2.1[1.0-4.5]).

**Table 1:** Test executed in the laboratories

	Maputo	Beira	Total
Test executed (n.)	1,515	1,556	3,071
Positive tests (n.)	76	315	391
Positivity rate (%)	5.0%	20.2%	12.7%
Rifampicin resistance (n.)	10	22	32
Rifampicin resistance rate (%)	13.2%	7,0%	8,2%
Rifampicin resistance prevalence (‰)	6.6	14.1	10.4

**Figure 1:**
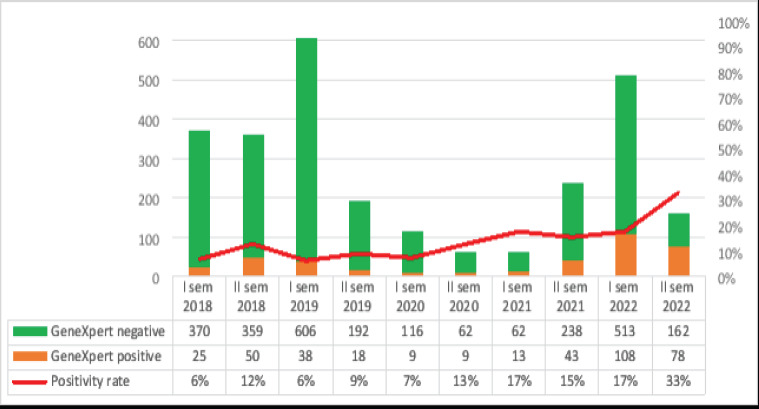
Temporal trends in TB tests executed

Figure 1 shows the temporal trend of laboratory activities, positive and negative results, and positivity rates. Temporal trends show a sensible reduction in 2020 and the first semester of 2021, and a recovery of diagnostic activity starting from the second semester of 2021. The decrement in laboratory activities in 2019 was mainly due to the Idai cyclone, which hit Beira and partially damaged the laboratory; the further reduction in 2020 was mainly related to a shortage in reagents provision due to COVID-19 and breaks in the supply chain in many African countries.

Despite the reduction in testing activity, positivity rates remained stable, with a slight increment starting in 2020.

## Discussion

Our field data confirm the trend described by the WHO about TB notification in Sub-Saharan Africa and Mozambique. All the high-burden countries show a reduction in TB notification rates in 2020 and 2021 compared with 2019 2. Mozambique is doing slightly better than other countries in the area, such as Lesotho which had a 38% reduction. Our data give an insight into this context. The increment in the positivity rate could be due to a better diagnostic capacity from the clinician requesting TB testing. COVID-19 could have improved the clinical skills of local personnel and reduced the number of useless TB tests.

Our data show different TB detection in Beira and Maputo. This factor could be explained by the usage of Xpert MTB/RIF Ultra in Beira since 2020. Xpert MTB/RIF Ultra in Beira seems to have higher sensibility compared with Xpert MTB/RIF^10^. However, the higher detection rate in Beira could reflect geographical differences in TB distribution in the country; we already described elsewhere such a geographical difference^11^. Probably both factors could be involved.

In our previous study conducted in 2017, rifampicin resistance was at lower levels (2,5%) than in the present analysis (8,2%)^11^. A similar growing trend is observed by analyzing data published in the Global Tuberculosis report in 2017 and 2021; the rifampicin resistance rate was 3,7% in the 2018 report and 6,2% in the last available report^2^,^12^. On the other side, the present data could also reflect an improvement in diagnostic capacity and training of clinical staff.

Many authors are concerned about the impact of COVID-19 on the global effort to reduce the TB burden^13^,^14^, and we agree. Shortage of laboratory reagents, particularly for TB diagnosis, was widely described in many regions^15^-^18^. However, our data highlight also eventual benefits coming from the COVID-19 experience in sectors of health systems. The pandemic represented a shock to health programs, however, in some cases, resilient systems reacted promptly and had some benefits^19^. As Trajman and colleagues discussed, COVID-19 had huge consequences for TB care all around the world, resulting in a 5-years set back in TB mortality^20^; however, CODIV-19 contributed also to some opportunities to reinforce TB control programs, as our data suggest.
